# Potential of Near-Infrared Spectroscopy for the Determination of Olive Oil Quality

**DOI:** 10.3390/s22082831

**Published:** 2022-04-07

**Authors:** Juan Francisco García Martín

**Affiliations:** 1 Departamento de Ingeniería Química, Facultad de Química, Universidad de Sevilla, 41012 Seville, Spain; jfgarmar@us.es; 2 University Institute of Research on Olive Groves and Olive Oils, GEOLIT Science and Technology Park, University of Jaén, 23620 Mengíbar, Spain

**Keywords:** chemometrics, olive oil, near-infrared spectroscopy, quality parameters

## Abstract

The analysis of the physico-chemical parameters of quality of olive oil is still carried out in laboratories using chemicals and generating waste, which is relatively costly and time-consuming. Among the various alternatives for the online or on-site measurement of these parameters, the available literature highlights the use of near-infrared spectroscopy (NIRS). This article intends to comprehensively review the state-of-the-art research and the actual potential of NIRS for the analysis of olive oil. A description of the features of the infrared spectrum of olive oil and a quick explanation of the fundamentals of NIRS and chemometrics are also included. From the results available in the literature, it can be concluded that the four most usual physico-chemical parameters that define the quality of olive oils, namely free acidity, peroxide value, K232, and K270, can be measured by NIRS with high precision. In addition, NIRS is suitable for the nutritional labeling of olive oil because of its great performance in predicting the contents in total fat, total saturated fatty acids, monounsaturated fatty acids, and polyunsaturated fatty acids in olive oils. Other parameters of interest have the potential to be analyzed by NIRS, but the improvement of the mathematical models for their determination is required, since the errors of prediction reported so far are a bit high for practical application.

## 1. Introduction

The International Olive Council defines olive oil as the oil obtained solely from fruits of the olive tree (*Olea europaea* L.), with the exclusion of oils obtained by solvents or by re-esterification procedures and any mixture with oils of another nature. As stated by this international intergovernmental organisation, the olive oils with the highest quality (so-called virgin oil oils) are those obtained ‘solely by mechanical or other physical means under conditions, particularly thermal conditions, that do not lead to alterations in the oil, and which have not undergone any treatment other than washing, decantation, centrifugation and filtration’ [1]. Virgin olive oils are classified, in turn, into extra virgin olive oils (EVOO), virgin olive oils (VOO), ordinary virgin olive oil, and lampante virgin olive oil [1,2], where EVOO is the olive oil of the highest quality. While the first three virgin olive oils are fit for consumption, lampante virgin olive oil must undergo processing prior to consumption.

Olive oil is regarded as one of the healthiest food oils due to its high content in triglycerides with unsaturated acids, mainly oleic acid, and its phenolic composition. The former is related to a decrease in LDL-cholesterol fraction, while the latter is responsible for the antioxidant properties and the bitter taste of olive oil. Triglycerides account for almost all the saponifiable fraction of the olive oil (roughly 98 wt.%). On the contrary, the phenolic compounds belong to the unsaponifiable fraction, which represents about 2 wt.% of total olive oil. The most representative phenolic compounds in olive oils are oleuropein and hydroxytyrosol. In addition to phenolic compounds, the unsaponifiable fraction is composed of a wide variety of compounds, such as triterpenic alcohols, α-tocopherol (vitamin E), γ-tocopherol, β-carotene (precursor substance of vitamin A and responsible for the yellow–orange colour of olive oil), phytosterols, sterols, hydrocarbons, chlorophylls (responsible for the green colour of olive oil), and volatile compounds responsible for the aroma of olive oil.

Due to the current popularity of the Mediterranean diet and its use in a wide range of different recipes, the nutritional value of olive oil is internationally recognized today. EVOO is mainly used as a salad dressing and food to be eaten cold due to its flavour and taste. The rest of the edible olive oils are used mostly for cooking.

The most common physico-chemical parameters that define the quality of olive oils are the free acidity (FA), the peroxide value (PV), and the absorbency in ultraviolet (at 232 and 270 nm). These three physico-chemical parameters, along with the organoleptic characteristics (odour and taste, defects, fruity attributes, and colour), are used by producers for the determination of the quality of virgin olive oils. Notwithstanding, the International Olive Council establishes additional quality criteria for the designation of olive oils (both edible and non-edible), namely moisture and volatile matter (wt.%), insoluble impurities in light petroleum (wt.%), flash point (°C), trace metal content (mg/kg of iron and copper), fatty acid ethyl esters content (mg/kg), and biophenols content (mg/kg) [1]. Generally, olive oil producers do not regard them as quality parameters, but as composition parameters. Additional physico-chemical parameters such as oxidative stability (h), chlorophyll and carotenoid pigment profiles, and the bitterness index are often included [2]. Regarding organoleptic characteristics, the absence (EVOO) or weak presence (VOO) of sensory defects and the presence of three positive attributes, namely fruitiness, bitterness, and pungency, must be evaluated by skilled tasters.

The determination of the aforementioned physico-chemical parameters is currently carried out in a laboratory using chemicals and generating waste, which is relatively costly and time-consuming. In addition, the online determination of the quality parameters of olive oil during the olive oil extraction process in olive mills is not possible using conventional methods. Among the various alternative, non-destructive methods for these analyses, the use of near-infrared (NIR) spectroscopy stands out. Its aim is to correlate the signal of the olive oils in the NIR spectrum with the quality parameters through the use of chemometrics. This article intends to show the state-of-the-art research and the actual potential of near-infrared spectroscopy (NIRS) for the analysis of olive oil, not only its main four quality parameters, but also other parameters of interest for the olive oil industry. For a better understanding, the following three sections include, in the following order, the fundamentals of NIRS, a description of the main features of the NIR spectrum of olive oil, and a brief explanation of what chemometrics is and how it is applied to NIRS, while the last three sections illustrate the results obtained by various authors on the quality parameters, other compounds of interest and sensory attributes, respectively.

## 2. Near-Infrared Spectroscopy (NIRS)

NIR spectroscopy is a vibrational spectroscopy, like Raman spectroscopy. A molecule absorbs NIR radiation (from 800 to 2500 nm) if the energy of the radiation corresponds to the energy difference between two vibrational levels and, in addition, a change occurs in the dipole moment of the molecule [3]. This is similar to what happens in the mid-infrared region. However, the bands of fundamental vibrations (Δ*n* = ±1, where *n* is the vibrational quantum number) do not appear in the NIR spectrum, while absorptions due to the overtones and combination bands are observed. The overtone bands are due to Δ*n* > ±1. Depending on the type of bonds, only the first (Δ*n* = ±2) and second (Δ*n* = ±3) overtones are likely to be observed. Combination bands occur only in polyatomic molecules and are due to simultaneous changes in the energy of two or more modes of vibration [3,4]. Therefore, the near-infrared spectrum is the result of the change in the molecular dipole moment during vibration. For example, the stretches of C=O in the CO_2_ molecule and of O–H in the water molecule, which are polar functional groups, have great absorption in the NIR spectrum [5]. Since the NIR spectrum of an analysed sample is the result of the combinations and overtones of the functional groups of its chemical constituents, the absorption peaks and bands found in the NIR spectrum are generally broad and weak. This makes NIRS more suitable for quantitative analysis than for compound identification (although NIRS can provide some information on functional groups). Hence, NIRS is regarded as a powerful analytical technique for the non-destructive, low-cost, rapid determination of compounds and parameters in food. Since NIR spectroscopy neither requires reagents nor generates waste, other advantages are providing a safe working environment and a huge potential for online measurement.

**Figure 1:**
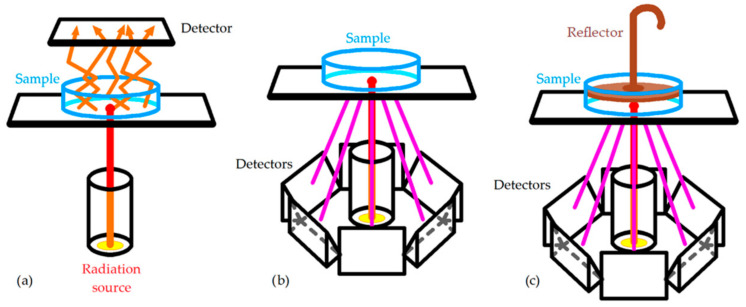
Main configurations to acquire NIR spectra: (**a**) transmittance; (**b**) reflectance; (**c**) transflectance [4].

An NIR spectrometer is composed of a radiation source (the most common is a tungsten–filament–fire halogen lamp with quartz window), a wavelength selector (generally a dispersive equipment), a sample holder, and a detector (generally built with semiconductors such as InGaAs and PbS). There are many sample holders depending on how the NIR spectrum is acquired [4]: transmittance, reflectance, and transflectance (Figure 1).

The use of cuvettes of different path lengths for transmittance and probes for transflectance is best for homogeneous liquids, while reflectance is generally used for solid, heterogeneous samples.

FTIR (Fourier-transform infrared) spectroscopy is an analytical technique generally used to identify functional groups in organic and inorganic compounds by obtaining their infrared spectra in the range of 2500–25,000 nm [6]. FTIR spectrometers acquire infrared spectra from solid, liquid, or gaseous samples in absorption, total, attenuated, and diffuse reflectance, and photoacoustic modes [6]. The raw signal is first Fourier-transformed by the equipment. FTIR spectrometers generate a unique type of signal called an interferogram that has all of the infrared wavelengths encoded into it [7]. Some authors regard the Fourier transform as a type of wavelength selector [4]. Although it is not the most common, FTIR spectroscopy can be applied to the NIR region, i.e., FTNIR spectroscopy, resulting in a faster NIR spectra acquisition with a higher signal-to-noise ratio than conventional NIRS [8]. Some works can be found in the literature on the use of FTNIR with olive oils for quantitative or discriminating purposes [9,10,11,12,13,14].

Since the 1980s, several works have addressed the determination of the main properties of olives of interest to the farmer. An industrial development of NIRS equipment to measure the internal properties of intact olives occurred about 15 years ago, so nowadays it is easy to find commercial equipment to non-destructively measure the moisture and fat content of olives, such as the OliveScan™2 and Olivia™ equipment (FOSS, Hilleroed, Denmark), the Luminar 5030 olive and olive paste analyser (Soluciones Integrales de Laboratorio, S.L., El Casar de Talamanca, Spain), and the NIT-38 olive analyser (NIR Technology Systems, Sidney, Australia). In addition, portable NIR spectrophotometers can be purchased for roughly EUR 6000 (e.g., Flame-NIR spectrometers, Ocean Optics, Inc., Orlando, FL, USA) and could be used at any stage of the olive oil production process. Although they have not been recognized as official methods by the International Olive Council, the determinations of fat content and moisture in olives by NIRS have been accredited as official methods by the pertinent authority of diverse countries. Thus, the accreditations 684/LE937 and 1335/LE2481 issued by the ENAC (Spanish Accreditation Bureau, Madrid, Spain) to various laboratories according to the criteria included in the UNE-EN ISO/IEC 17025:2017 standard [15], officially allow the determination of total fat and moisture in intact olives by NIRS following an internal method based on the manufacturer’s method FOSS for the Olivia^TM^ equipment (FOSS, Hilleroed, Denmark). Notwithstanding, and despite the large available literature, such industrial development does not exist for the measurement of the quality parameters of olive oil.

**Figure 2:**
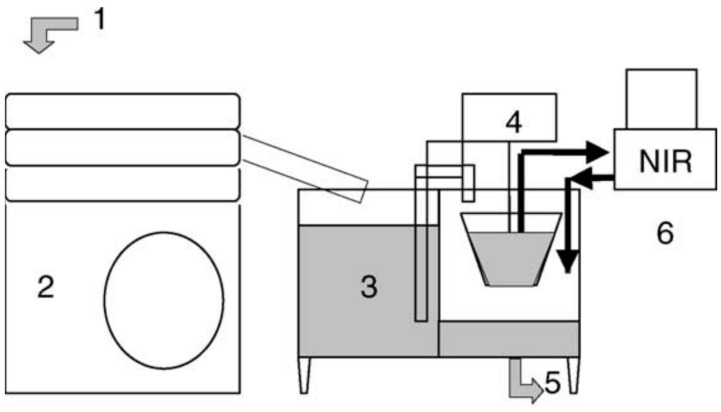
Schematic diagram of the proposed NIRS sensor in the last pass in the olive oil extraction process: (1) oil from horizontal centrifuge decanter; (2) vertical centrifuge for oil clarification; (3) tank for oil sedimentation; (4) continuous oil weigher; (5) to oil storage container; (6) NIRS equipment [18].

At the beginning of the twentieth century, several works have addressed the use of NIRS to determine the olive oil quality’s parameters at different points in the oil extraction process in olive mills [16,17,18]. Thus, NIRS equipment was installed on an olive oil production line, in order to take samples at the exit of the clarifying centrifuge and from the hopper where the oil is continuously weighed, as shown in Figure 2 [18]. Furthermore, NIRS has been applied to design a quality control system for the identification of adulterated olive oils with other oils such as sunflower oil, corn oil, and raw olive waste [19], and to the differentiation of olive oils that belong to different denominations of origin [20]. Comprehensive reviews on these latter topics can be found elsewhere [21].

Once experts in the olive oil production process have been consulted, three points within the process, which can be found in Figure 2, seem to be the most suitable for the sampling of olive oils and the on-site determination of their quality parameters by NIRS. The first is at the exit of the horizontal decanter (2- or 3-outlet decanter). However, the samples taken at this point would be more intended for experimental purposes and the enhancement of the process (assessment of temperature of the olive oil in the decanter, etc.), and the values of the quality parameters could not match those of the oil once bottled. The second and third would be at the exit of the vertical centrifuge for oil clarification (or the sedimentation tank if available in the olive mill) and at the olive oil storage containers, respectively. Nevertheless, considering that NIRS is a rapid, non-destructive, technique that requires minimal sample preparation (no reagent) and relatively small amounts of a. sample (a few mL of olive oil), experts consider that the most suitable location for the NIRS equipment would be at the bottling plant. In this way, after filling a bottle with olive oil, a small sample would be immediately taken and its NIR spectrum acquired, which would provide the actual values of the quality parameters of the olive oil contained in the bottle. This would also speed up and make the olive oil labelling process more precise, as long as the International Olive Council (or the national quality bureaus) accepts NIRS as an official method for the determination of the olive oil’s quality parameters.

However, such types of online proposals have not been, or have been installed only to a limited extent, in olive mills. This is because the development of robust mathematical models is the key to the industrial application of NIRS for online monitoring. These models, previously obtained by using chemometrics, could be the starting point for designing and installing an online tool for the determination of the quality parameters of olive oil on process lines at olive mills, but a full-scale application requires a huge number of samples, not only from the different varieties of olives that the olive mill works with but also over several harvestings in order to develop mathematical models that can be used in a production context. In addition, the chemometric tools of NIRS equipment should provide self-learning model calibration systems. That is to say, the just-acquired spectra directly from the oils in the process line should be automatically included in the calibration data set to strengthen the models by expanding the data sets over time [16]. In this sense, FOSS annually updates the calibration models of their NIRS equipment to measure properties in intact olives and olive pastes, and customers have to pay to update their equipment if they apply for it.

## 3. Near-Infrared Spectrum of Olive Oils

**Figure 3:**
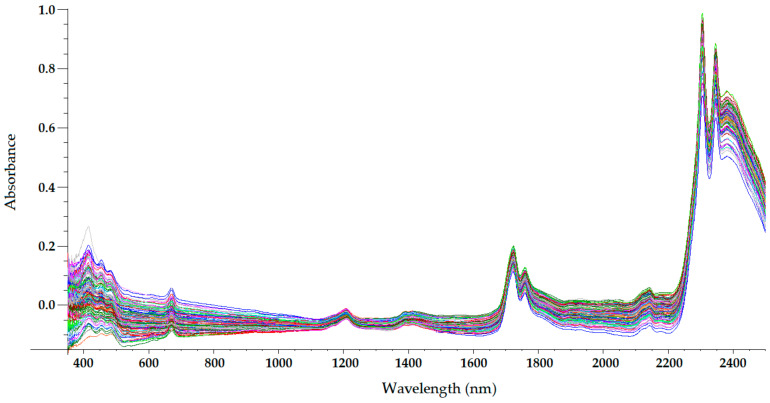
Mean-normalized visible/NIR spectra of 127 olive oils obtained with 0.5-mm path-length quartz cuvette [22].

**Figure 4:**
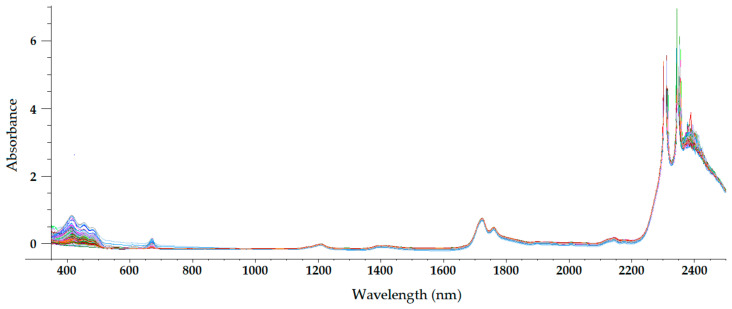
Mean-normalized visible/NIR spectra of 127 olive oils obtained with 2-mm path-length quartz cuvette [22].

**Figure 5:**
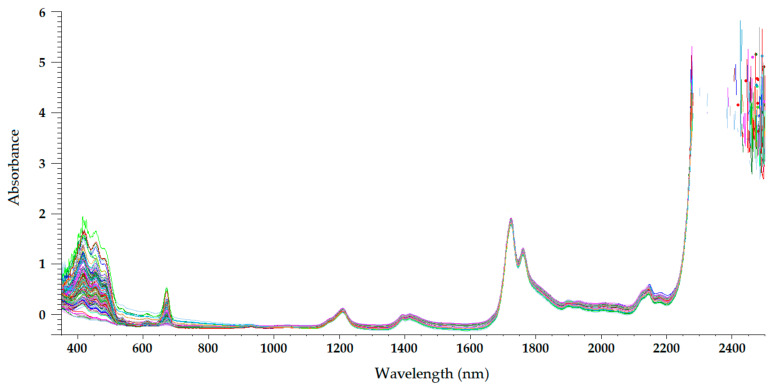
Mean-normalized visible/NIR spectra of 127 olive oils obtained with 5-mm path-length quartz cuvettes [22].

**Figure 6:**
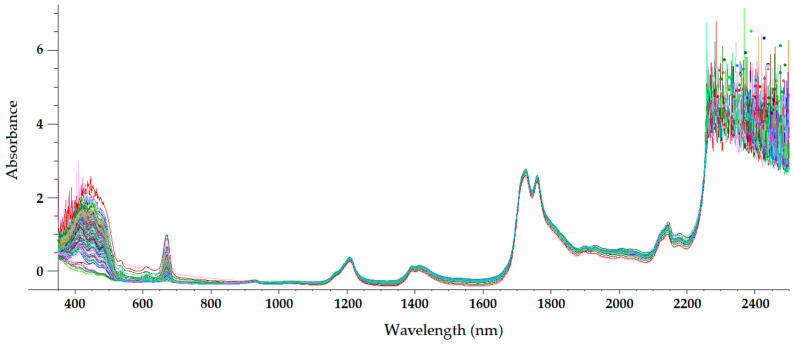
Mean-normalized visible/NIR spectra of 127 olive oils obtained with 10-mm path-length quartz cuvette [22].

The sample temperature has a great influence on the NIR radiation that it reflects and absorbs, which makes temperature a parameter of paramount importance in NIRS. For olive oils (and other oils), a sample temperature of approximately 32 °C is usually chosen [22,23,24,25,26,27,28]. At this temperature, olive oil is a homogeneous liquid, with a non-important loss of volatile compounds occurring. Therefore, the only sample preparation required in NIRS is heating and maintaining olive oil at that temperature during spectrum acquisition. On the other hand, near-infrared radiation penetrates deeper into organic samples than other electromagnetic radiations, such as ultraviolet (UV), visible, far-infrared, and mid-infrared radiations [29]. Therefore, the optical path length chosen when acquiring NIR spectra has a significant influence on the radiation absorption intensity at different wavelengths. Figure 3, Figure 4, Figure 5 and Figure 6 show the visible-NIR spectra of 127 olive oils from the variety ‘Picual’ acquired using a Labspec Pro 350-2500P visible/NIR spectrophotometer (Analytical Spectral Devices Inc., Boulder, CO, USA) equipped with three detectors and an operating in transmittance mode. This equipment was used for the spectral acquisition of olive oils in the range 350–2500 nm using quartz cuvettes with different path lengths (from 0.5 to 10 mm) as sample holders. The reflectance was transformed into absorbance. As shown in these figures, the use of cuvettes with higher path lengths for spectral acquisition leads to higher absorbance in the NIR region, thus providing smoother NIR spectra that are more suitable for further building mathematical models for the determination of quality parameters [22]. In this sense, small differences in path length (0.2 and 0.5 mm) when acquiring the spectra of South African EVOO in the transflectance mode with quartz cuvettes as sample holders have been reported not to have a significant effect on regression model performance [9].

The NIR spectrum of olive oil has previously been described in the literature by various authors [21,22,27,29,30]. In fact, the NIR spectrum of olive oil is quite similar to that of triglycerides, as olive oil is mainly composed of triglycerides [31]. What is more, as triolein is the main triglyceride and therefore the major component of olive oil, the highest absorption band in the NIR spectrum of olive oil is the same as that of the triolein spectrum, which is observed at 1725 nm [29]. The two regions of the NIR spectrum that are of great importance [31] can be clearly observed in Figure 3, Figure 4, Figure 5 and Figure 6. One is the absorption band near 1720 nm, which is related to the first overtone of the C-H vibration of several chemical groups such as –CH_3_, –CH_2_ and =CH_2_, and the other is the absorption peaks at 1660 and 2145 nm, which are related to the C-H vibration of *cis*-unsaturation. When the degree of *cis*-unsaturation increases, the absorption peak at 1725 nm (*cis*-C18:l) shifts to lower wavelengths, i.e., to 1717 nm and 1712 nm for *cis*-C18:2 and *cis*-C18:3, respectively [31]. The high adsorption peak at 2145 nm makes the main peaks related to saturated and *trans* fatty acids, usually observed at 2128 and 2131 nm, respectively, hardly noticeable in the olive oil spectrum [29]. Wavelengths close to 1800 nm have also been related to the saturated fatty acids [29]. Finally, a broad absorbance band can be observed at 1210 nm as a result of second overtones of C–H and CH=CH– stretching vibrations [27].

Besides the bands and peaks corresponding to molecules that compose the fatty acids of the olive oil, a broad band at 1400 nm and a broader one at around 1950 nm are also observed in these figures. These bands have been related to the presence of water, to be specific to its first overtone, and to a combination band, respectively [25].

As observed in Figure 3, Figure 4, Figure 5 and Figure 6, the absorbance in the 2300–2500 nm region is out of the range of the detector used (a holographic fast scanner InGaAs detector, cooled at 25 °C, and coupled with a high-order blocking filter) when increasing the path length of the cuvette (lower radiation transmission and therefore higher absorbance by the olive oil). This problem has also been reported when disposable borosilicate vials were used for the spectral acquisition of olive oil between 400 and 2500 nm in the transmittance mode [32]. This problem was attributed to the high absorbance of this material. For this reason, quartz cuvettes are the most used and appropriate sample holders for NIRS, since quartz does not absorb radiation in the NIR region [21]. However, since neither of the two regions of major importance in the NIR spectrum of olive oil falls in this region, the absorbance at wavelengths between 2200 and 2500 nm can be discarded when working with olive oil NIR spectra without losing important information on the sample.

Regarding the visible spectrum, it is sometimes used together with the NIR spectrum for the determination of olive oil’s quality parameters. There are three main absorption peaks of olive oil in the visible spectrum. The first is found at 415 nm (dark blue coloured light) and is related to carotenoids, pheophytin *a*, pheophorbide *a*, and pyropheophytin *a* [33]. The second absorption peak can be observed at 450 nm (blue light), which is characteristic of carotenoids [33]. The third absorption peak is found at 670 nm, and is related to chlorophylls [27]. It is worth noting that the two former peaks (between 350 and 500 nm) were easier to differentiate with the 0.5-mm and 2-mm quartz cuvettes (Figure 3 and Figure 4, respectively) than with the 5-mm path-length cuvette (Figure 5). They could not be clearly distinguished using the 10-mm path-length cuvette (Figure 6), which could indicate that increasing the path length when working in the transmittance mode reduces the quality of the visible spectrum of olive oil. This is contrary to what was found in the NIR spectrum.

The features of the visible and NIR spectra of olive oil have been exploited in different ways. For example, the absorbances in the 470–690 nm, 1145–1265 nm, and 1355–1500 nm visible/NIR ranges have been related to olive pomace oil, so these spectral ranges have been used to determine the amount of olive pomace adulterating EVOO with a low standard error of prediction (SEP = 3.27 wt.%) [5]. Besides, two minor carbonyl absorptions at 1894 and 1930 nm have been used to assess the authenticity of EVOO based on the ratio of absorption intensity at these wavelengths, which are related to the loss of volatiles from EVOO, and therefore to the loss of quality of olive oils [11]. On the other hand, the use of wavelengths in which the absorption of NIR radiation is related to the structure of fatty acids (aliphatic chains), and therefore responsible for the free acidity of olive oil, resulted in more reliable mathematical models for the determination of free acidity in edible olive oils [22].

## 4. Chemometrics Coupled with NIRS

Chemometrics is defined as ‘the science of relating measurements made on a chemical system or process to the state of the system via application of mathematical or statistical methods’, according to the International Chemometrics Society [34]. It started to be applied to spectroscopic data about five decades ago. Chemometrics coupled with NIRS can be defined as the application of statistics and mathematical models to extract the desired information from the NIR spectra. The NIR spectra of olive oils are difficult to interpret since they are the result of overlapped overtones and combination bands, which can contain different baselines or noise. The combination of NIRS and chemometrics provides calibration models for olive oil spectra analysis and both classification and discrimination tools. Chemometrics coupled with NIRS are also suitable to handle the dimensional overload, collinearity, spectral interferences, and spectral noise on olive oil NIR spectra. To do this, several specific software has been developed, such as The Unscrambler (CAMO Software AS, Oslo, Norway) or the Chemometrics Toolbox (Eigenvector Research, Inc., Manson, WA, USA) for MatLab (The MathWorks, Inc., Natick, MA, USA), which allow obtaining results with great precision, speed, and comfort.

To speed up data evaluation and to increase the precision of the mathematical models, pre-treatments are generally applied to raw spectra, consisting of classical methods for spectral normalization, smoothing, and differentiation [35,36]. Spectra pre-treatments include data spectra derivatization, normalization, baseline correction, standard normal variate, mean centring, Savitzky and Golay smoothing, first and second derivatives and multiplicative scatter corrections [8,36,37,38]. The use of spectra pre-treatments, which at first is an advantage for the use of NIRS for the determination of quality parameters of olive oil, can result in a huge hindrance to the implementation of NIRS for online monitoring or on an industrial scale. For example, when applying a normalization (generally maximum normalization or mean normalization) to olive oil spectra, all available spectra are selected for that normalization, and the normalized spectra are subsequently used to build a calibration method for the determination of one or more olive oil properties via chemometrics. As mentioned above, the chemometric tools coupled with NIRS should provide self-learning calibration models. That is to say, spectra acquired later (e.g., olive oils from next harvestings) must be included in the calibration data set to expand data sets and strengthen models over time [16]. The problem is that the current set of spectra has already been normalized. The new added spectra cannot be normalized in the same way. At most, all the spectra (old and newly acquired) could be normalized together, but this normalization would be different from the normalization done with the old spectra, thus affecting the later selection of outliers, the developed calibration model, etc. As a result, this kind of pre-treatments would be difficult to implement for an online measurement of olive oil’s quality parameters during olive oil extraction at the olive mills.

For olive oils, chemometrics coupled with NIRS are generally used for oil classification (including adulterations) or property quantification. To do this, there are mathematical algorithms that explore the correlation structure within a single data block. For olive oil classification, unsupervised pattern recognition such as principal component analysis (PCA) and supervised pattern recognition such as partial least squares (PLS) combined with discriminant analysis (DA) is the most used chemometric technique [36,39]. Many works can be found in the literature for the detection of adulteration in olive oils using NIRS. Thus, PCA has been applied to detect corn, sunflower, or raw olive residue oils in the range 0–30 wt.% in VOO and EVOO [19], to detect between 5 and 50 wt.% sunflower, soybean, and sesame oils in VOO [40], and to detect corn, sunflower, soybean, and canola oils in EVOO, with lower limits of adulteration detection of approximately 20, 20, 15, and 10 wt.%, respectively [41], all of them in the laboratory. The good results obtained in the determination of adulteration in EVOO using PCA and NIRS have led to testing the use of portable NIR spectrometers, which could provide in situ information on adulteration. In this sense, it was proven that the use of PCA and a portable spectrometer, which collected spectra in the range 908−1676 nm, resulted in a reliable tool to identify, classify, and quantify the content of different vegetable oils (canola, corn, soybean, and sunflower oil) in EVOO at a confidence level of 95% [42]. On the other hand, PLS-DA has been applied, for example, to detect corn, hazelnut, soya, and sunflower oils in olive oils [43]. Furthermore, PCA and PLS-DA of olive oil NIR spectra have also been applied to predict the geographical origins of olive oils. For example, 57 EVOO were successfully classified according to their geographical origin (Chianti Classico or Maremma) using different pre-treatments and chemometric methods; among them, PCA stood out [44]. Both PCA and PLS-DA were used to discriminate between 135 VOO (10 commercial VOO and 125 VOO from 5 French Protected Designation of Origin) based on their NIR spectra features [45].

In order to correlate the NIR or visible/NIR spectra of olive oils with the quantifiable parameters of interest, multivariate calibration methods are applied, namely multiple linear regression (MLR), principal component regression (PCR), and partial least squares (PLS) regression. Regarding the determination of olive oil quality parameters by NIRS, few papers can be found in the literature that apply MLR or PCR [46]. In contrast, in almost all the published articles available in the literature dealing with NIRS and the determination of the quality parameters of olive oil, the building of predictive models is based on PLS regression [9,18,22,23,24,25,28,30,46,47].

The parameter of interest (acidity, peroxide value, etc.) must be previously analysed by the traditional, official method (i.e., the reference method according to the International Olive Council standard), to use the obtained values for building the mathematical model with which this parameter will be measured in the future by NIRS. That is to say, the spectra of the olive oils will be correlated with the values of the parameter of interest measured with the reference method.

For a quick explanation of these three regression methods, *R* will be defined as the matrix *i* × *j* of the absorbances of the *i* samples at the *j* wavelengths of the NIR spectrum and *C* as the matrix *i* × 1 of the different values of the olive oil’s parameter to be analysed by the NIRS for each sample.

Multiple linear regression (MLR) is a method that directly establishes a linear combination of the variables of *R* (absorbances at different wavelengths) that reproduces the values of *C* (values of the olive oil’s parameter measured by the reference method) minimising the error (Equation (1)).

*C* = (*R* × *S*) + *E* (1)

where *S* stands for the matrix of coefficients that, multiplied by the values of *R*, provides the matrix of values of the analysed parameter (*C*), and *E* is the residual error matrix [4,38,48]. This method is the least used and is applied when the number of samples is greater than the number of variables [37].

Principal component regression (PCR) is a method in which the matrix *V* of the principal components (PC) of *R* is first determined. The first principal component (PC1) is the vector in the column space of *R* that describes the maximum amount of variation within the spectra of the olive oils. The second principal component (PC2) describes the maximum residual variation not described by PC1, and so on. The minimum number of PC that minimises the information not explained is selected. Then, the projection of *R* in *V* is performed, thus obtaining the matrix of scores *U* (Equation (2)). Finally, a linear combination of *U* provides the values of *C* that minimise the error (Equation (3)).

*U* = (*R* × *V*) (2)

*C* = (*U* × *S*) + *E* (3)

Thus, to determine by NIRS the value of the parameter *C* of an olive oil sample, different from those used for PCR, the scores matrix *U*_unk_ is obtained from the absorbance matrix *R*_unk_ by multiplying it by the matrix of principal components *V*. Then, the value of the parameter *C* of that sample is obtained by introducing *U*_unk_ in Equation (3) [4,38,48].

Finally, partial least squares (PLS) regression is the most used method, and the most suitable when the number of samples is smaller than the number of variables [37,49]. Furthermore, PLS regression provides a better approach to quantitative modelling than MLR, because the correlations among the noise in *R* are more realistic [49].

In this method, the projection of both *R* and *C* is performed in the space *V* defined by the PC, i.e., the projection of *R* in *V* provides a matrix of scores *U*, and the projection of *C* in *V* leads to the score matrix *T* (Equation (4)).

*T* = *C* × *V* (4)

From these score matrices, the following equations are obtained:

*R* = (*U* × *P*) + *E* (5)

*C* = (*T* × *Q*) + *F* (6)

*T* = (b × *U*) + *G* (7)

where *P* stands for the loadings matrix of *R*, *Q* is the loadings matrix of *C*, b is a constant and *E*, *F* and *G* are the residual matrices (error matrices). The ideal situation to relate *R* to *C* is when *U* and *T* are very similar. That is, b should be close to 1.

Therefore, for an olive sample not used in the PLS regression of which the value of the parameter *C* is unknown, the scores matrix *U*_unk_ is calculated from the values of its NIR spectrum matrix *R*_unk_ using Equation (2), which in turn will allow one to obtain the scores matrix *T*_unk_ using Equation (7). Once *T*_unk_ has been calculated, the matrix *C*, that is, the parameter of olive oil to be calculated by NIRS, is obtained [4,38,48].

Once the calibration model is built by MLR, PCR, or PLS, it is necessary to assess its predictive capacity when applied to samples not used in the calibration process. In other words, validation is necessary to determine the extent to which the results obtained can be extrapolated from samples different from those used to build the calibration method, so the model can be used to determine the parameter desired by NIRS in olive oils from, for example, future harvestings [4,38]. Therefore, in the research papers available in the literature for the determination of the olive oil’s quality parameters by NIRS, the samples are usually divided into calibration and validation sets, so that some of the well-characterized samples are reserved to validate the accuracy of the model. In most cases, the calibration set is made up of two thirds of the samples and the validation set of the remaining third, the selection from the samples of each set being random [9,23,24,25,47]. Other authors have selected one out of four olive oils for the validation set, the remaining olive oils forming the calibration set [30]. However, this does not guarantee a good spread of spectral variability within both sets, so samples for the calibration set should not be selected primarily as a function of their number, but rather for their variability [34]. This means that increasing the number of samples for the calibration set does not always result in a more accurate and robust model.

The simplest solution is to distribute samples uniformly within both calibration and validation sets, taking into account the highest and the lowest values of the parameter of interest of olive oil (measured in the samples with the reference method) to be analysed by NIRS. However, with this solution, only the variability in the analysed parameter is distributed, while the distribution of the variability in the spectral information remains uncertain. The most used method in NIRS that takes into account the variability among spectra is the Kennard–Stone method [50]. The Kennard–Stone algorithm is applied to the spectra (not to the values of the parameter of interest). To select the samples for the calibration set, the algorithm starts by searching for the two samples with the largest Euclidean distance. The following samples for this set will be those that maximise the Euclidean distance from previously selected samples, and so on. This will guarantee that all the variation within the spectral information is contained in the calibration sample set. The Kennard–Stone method has been applied, for example, in the determination of the acidity of olive oil by NIRS [22]. When there is not a validation set of samples, an internal validation method is used, which uses the same samples of the calibration set to validate the mathematical model. The most commonly used internal validation method is full cross-validation (CV). It consists of creating models using all samples except one and validating the model with the excluded sample (leave-one-out method). Therefore, *n* calibration models are built from *n* samples. The standard error of cross validation is obtained from the arithmetic mean of the error values obtained in the *n* models [51].

The robustness of the PLS calibration models is usually evaluated by the multiple correlation coefficient of calibration (r^2^_c_), while their ability to predict the parameter of interest is assessed by the standard error of prediction (SEP) or the root mean square error of prediction (RMSEP). Both SEP and RMSEP describe the error between the results from the reference method and the results from the NIRS equipment for a set of unknown samples not used for the building of the PLS calibration model. SEP is related to the precision of the model, while RMSEP is related to its accuracy. If the samples were not divided into calibration and validation sets, then the standard error of cross-validation (SECV) or the root mean square error of cross-validation (RMSECV) is used instead.

Generally, an ideal PLS model should have a very high r^2^_c_ and a value of SEP close to the standard error of laboratory (SEL) of the reference method. The closer SEP is to SEL, the greater the precision of the PLS model and the probability of this to provide roughly the same values of the parameter of interest as the reference method. Table 1 summarises the criteria proposed by Shenk and Westerhaus to assess the statistical results of the PLS calibration models and their validations.

Usually, the larger the number of samples used for building the calibration model, the better the predictive capacity of the model, and the smaller the error of prediction. There is not a rule about how many samples should contain the calibration set, but it is informally accepted that at least 100 samples should be used for building the calibration models. However, this number of samples is not mandatory and robust calibration models can be built with fewer samples.

The number of principal components used in the PLS model is also related to the performance of the model. The lower the PC number, the better. Normally, the minimum number of PC that maximises the explained information of the PLS model is chosen.

The performance of the PLS models is also assessed by the ratio of performance to deviation (RPD), also called the residual predictive deviation. This parameter is defined as the ratio of the standard deviation (σ) of the reference data from the validation set to the SEP. It is assumed that PLS models with RPD values higher than 3 can be suitable for routine analysis. This parameter is very popular in the literature but, in the opinion of the author, is a tricky parameter. For example, consider that a parameter of food must have a value less than 1 unit to be accepted for human consumption. Imagine that the samples to validate a PLS model have values in this parameter from 0 to 10 units used, the average value of the samples is 5 units, the standard deviation is 2.5 units, and the achieved SEP is 0.5 units. As RPD is defined as σ/SEP, then RPD = 2.5/0.5 = 5, the method will thus be regarded as a method of great precision. In the opinion of the author, a new PLS model for determining a parameter in food (based on the data obtained from a reference method) which must be less than 1 unit and of which the SEP is 0.5 units (i.e., the average difference between the values provided by the new method and the reference method is 0.5 units), is not a very good one, regardless of its acceptable RPD value. The next section will provide some examples on this matter.

Unfortunately, although PLS regression is a powerful tool for building calibration models from NIR full-spectrum, even noise, background and uninformative wavelengths have the possibility of being included in the models [22,37,53]. In the literature, several mathematical methods can be found to remove these wavelengths and only let those wavelengths that actually contribute to the PLS model remain, such as Monte Carlo uninformative variable elimination (MCUVE) [22,53,54,55], moving window variable importance in projection [56,57], the successive projections algorithm (SPA) [22,55,58], etc. Other authors perform the selection of the spectral variables involved in the models by consecutive cycles, removing those which contribution to the model (regression coefficient) close to zero in each cycle [24,28].

Another interesting option to improve the performance of PLS models is to remove outliers. If the prediction sample is inconsistent with the calibration data, it is regarded as a prediction outlier [59]. They can be removed manually or by applying multivariate outlier detection methods. However, wavelength selection and outlier removal must be carefully performed or avoided at early stages due to the risk of eliminating important spectral information related to the quality parameter of interest. As mentioned above, NIRS equipment should provide self-learning model calibration systems, i.e., spectra from new samples (new harvestings, different geographical origins, etc.) should be automatically included in the calibration data set to strengthen the PLS models by expanding the data sets over time [8]. Only once a robust PLS model is created for determining a quality parameter from hundreds (or thousands) of olive oils of different varieties, harvestings, geographical origins, etc., should the selection of variables and removal of outliers be performed, and the resulting PLS validated with new samples from next harvestings, etc.

## 5. Determination of Olive Oil’s Quality Parameters by NIRS

### 5.1. Free Acidity (FA)

The acidity value or free acidity of an oil is a measurement of its free fatty acids content, which is released from the hydrolysis of oil triglycerides by lipolytic enzymes. These enzymes are normally present in the seed and pulp cells of olives. When the integrity of the fruit is damaged, the enzymes react with the oil contained in vacuoles. Unhealthy, damaged, or bruised olives, along with unsuitable storage conditions, are responsible for olive oils with high acidity values [2].

FA is expressed as a percentage of grams of oleic acid per 100 g of oil. The conventional determination of FA is carried out in the laboratory using chemicals according to the Official Methods of Analysis of the European Commission [60], being relatively costly and time-consuming. Briefly, the method consists of placing a few grams of olive oil into wide-mouth Erlenmeyer flasks, along with an ethyl alcohol:ethyl ether solution (1:1 *v*/*v*) and a few drops of phenolphthalein, to neutralize the free fatty acids with NaOH until pink in colour [22].

Olive oils with FA greater than 2% are not regarded as fit for consumption and must be refined prior to consumption [1]. With regard to edible olive oils, according to the European Regulation, the maximum levels of free acidity for EVOO and VOO are 0.8% and 2%, respectively, while the FA threshold for olive oils (blends of refined olive oil and VOO fit for consumption) and olive pomace oils (obtained by treating olive pomace with solvents) is 1%.

The estimation of FA by NIRS has been previously assayed by several authors (Table 2), achieving significantly good results in general. Thus, the average FA values for ‘Arbequina’ and ‘Picual’ olive oils were 0.49 ± 0.01 and 0.33 ± 0.00, respectively, by means of the reference method [60], while the average values were 0.54 ± 0.15 and 0.37 ± 0.16, respectively, using the 1100–2500 nm NIR spectrum [18]. For the calibration set, these authors used olive oils with acidity between 0.12 and 15.1%, while for the validation set, the olive oils had FAs ranging between 0.16 and 12.2%. Using 15 PC, these authors achieved a R^2^_cal_ of 0.998 and a SEP of 0.16% (Table 2). This error was very close to the SEL estimated by the authors (0.1%), which accounts for the robustness of the PLS model. As illustrated in Table 2, by reducing the free acidity range of olive oils for creating the PLS models, lower SEP was achieved. It is worth noting that an SEP of 0.35% [27] and an RMSEP of 0.34% [61] led to an RPD greater than 3. As indicated in the previous section, these values of RPD could make one think that these PLS models have good precision. However, these predictive errors seem to be slightly too high to be suitable for measuring FA or discriminating between edible olive oils, of which the maximum allowed FA is 2% (0.8% for EVOO). Besides, these errors are much higher than SEL for the reference method reported by several authors: 0.1% [18], 0.048% [22], and 0.032% [9]. In Table 2, it can be observed that PLS models with low RPD such as [47] showed low prediction errors, because the FA range chosen to build the calibration model was more appropriate.

**Figure 7:**
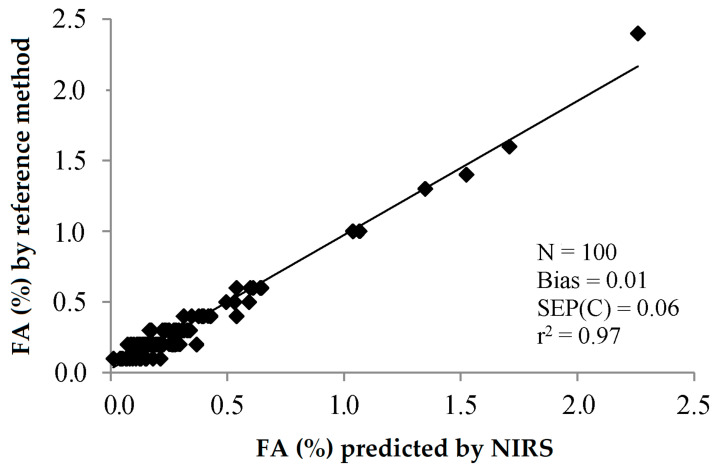
Validation exercise for the determination of the free acidity of olive oil by PLS-NIRS using all the wavelengths between 400 and 2500 nm [30].

All in all, it can be concluded that the free acidity of olive oils can be measured by NIRS with great precision. This precision can be graphically observed when plotting the predicted values against the FA measured by the reference method (Figure 7).

**Figure 8:**
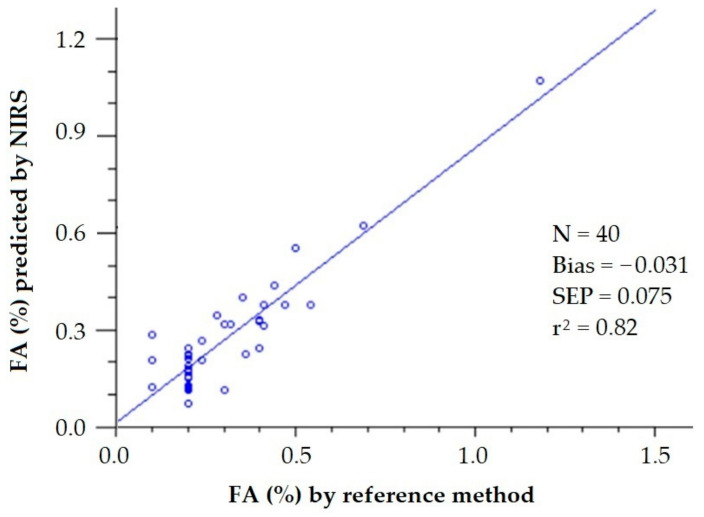
Validation exercise for the determination of the free acidity of olive oil by PLS-NIRS using all the wavelengths between 800 and 2200 (FA values retrieved from [22]).

**Figure 9:**
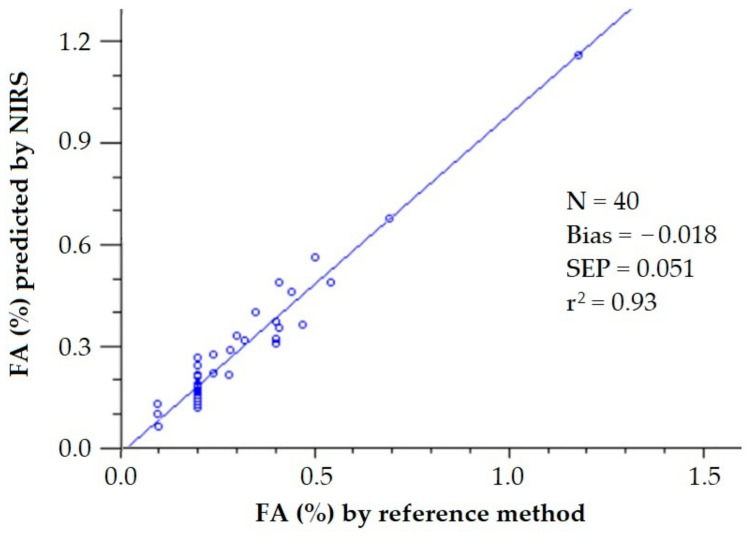
Validation exercise for the determination of the free acidity of olive oil by PLS-NIRS using 85 wavelengths from the NIR spectrum (FA values retrieved from [22]).

In a previous work, the elimination of noise and uninformative spectral variables affecting a PLS model for the determination of FA of olive oils by NIRS was assayed by the Monte Carlo uninformative variable elimination (MCUVE) method and the successive projections algorithm (SPA) [22]. When using the 1401 wavelengths from 800 to 2200 nm, the achieved SEP was 0.75%. The PLS model built with the 314 wavelengths selected by MCUVE led to SEP = 0.064%, while the MLS model built with the 85 wavelengths selected by SPA was 0.051%, quite close to the SEL (0.048%) reported by the author. This improvement in the goodness in the prediction can be visually observed when plotting the FA values predicted by the PLS calibration model built with the full NIR spectrum (Figure 8) and by the MLS calibration model built with the 85 selected wavelengths by SPA (Figure 9) against the FA values obtained using the reference method.

Interestingly, only 12 of the 80 wavelengths selected by SPA were among the 314 wavelengths selected by MCUVE. This accounted for the difficulty of interpreting the NIR spectra and PLS models obtained from them. It was found that most of the selected wavelengths by MCUVE were related to the main NIR absorption bands of free fatty acids. On the contrary, most of the wavelengths selected by SPA were correlated with triacylglycerols [22]. A tentative assignment of the wavelength ranges selected by each method has been carried out by the author (data not previously published) and illustrated in Table 3. Several years later, the author tried to predict the FA of waste cooking oils with the PLS models obtained for olive oils, with the wavelengths selected by MCUVE and SPA. The statistics were quite poor (hence the prediction error was very high), which could be due to impurities in the waste cooking oils used or the premature removal of wavelengths when creating the PLS models. In the end, the author had to build a specific PLS model to determine the free acidity of waste cooking oils by NIRS [64].

Finally, some works can be found in the literature on the determination of FA in other IR spectral regions different from the NIR range. For example, the use of FTIR spectroscopy in the infrared spectral region from 5800 to 6075 nm and the wavelength 3308 nm resulted in an R^2^_cal_ of 0.99 and a root mean square error of cross-validation of 0.0107% [13]. However, the calibration model was built with solely a set of 15 samples with FA between 0 and 1%, which were prepared by the gravimetric addition of oleic acid to deodorised olive oil.

### 5.2. Peroxide Value (PV)

Peroxides are the primary products of the oxidation of olive oil. The peroxide value is a measure of the total peroxides in olive oil expressed as mEq O_2_/kg oil, and therefore a major quality guide. EVOO and VOO cannot exceed the maximum value of 20 mEq O_2_/kg, the limit fixed by the International Olive Council [1].

The reference method consists of dissolving the oil sample in acetic acid and chloroform, adding potassium iodide and subsequent titration with sodium thiosulphate of the liberated iodine [60]. The precision of the reference method was determined from the results of collaborative tests by the International Olive Council [28], the reproducibility and repeatability coefficients of variation being 7.1% and 1.9%, respectively, for EVOO, and 13.8% and 3.4% for ordinary olive oils. The standard error of laboratory was reported to be 1.41 meq O_2_/kg [9].

Table 4 illustrates the errors of prediction of PV by NIRS achieved by several authors. As can be seen, most of the SEP values are close to the reported SEL, even though the RPD values are not too high. Therefore, it can be concluded that PV is another olive oil’s quality parameter that can be predicted by NIRS.

**Figure 10:**
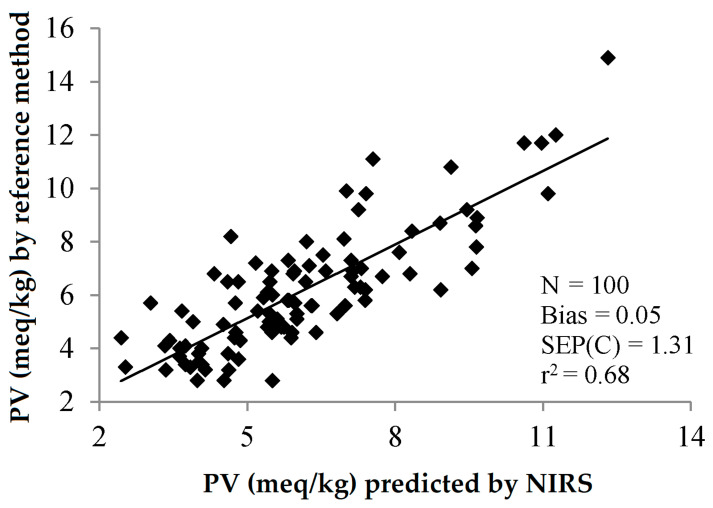
Validation exercise for the determination of the peroxide value of olive oil by PLS-NIRS using all the wavelengths between 400 and 2500 nm [30].

However, when comparing Figure 7 with Figure 10, it can be observed that the precision of the determination of the peroxide value by NIRS seems to be lower than that of the free acidity.

### 5.3. K270 and K232

The determination of UV-specific extinctions permits an approximation of the oxidation process in unsaturated oils. At 232 nm, primary oxidation products show an absorption (conjugated dienes) that increases due to the defective storage of olive fruits or faulty oil extraction. Secondary oxidation products, such as carbolynic compounds (aldehydes and ketones), are detected at 270 nm, indicating an advanced oxidation process. The maximum permitted values are 2.5 for K232 and 0.20 for K270 [1]. The extinction coefficients K232 and K270 are measured by UV spectrophotometric analysis at the specific wavelengths of 232 and 270 nm and are expressed in absorbance units (AU). Notwithstanding, their determination has been assayed by NIR and visible/NIR spectroscopy (Table 5 and Table 6).

**Figure 11:**
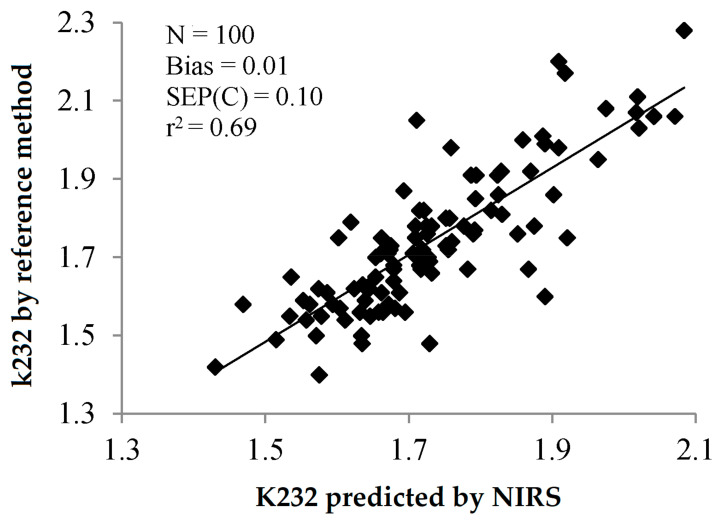
Validation exercise for the determination of K232 of olive oil by PLS-NIRS using all the wavelengths between 400 and 2500 nm [30].

**Figure 12:**
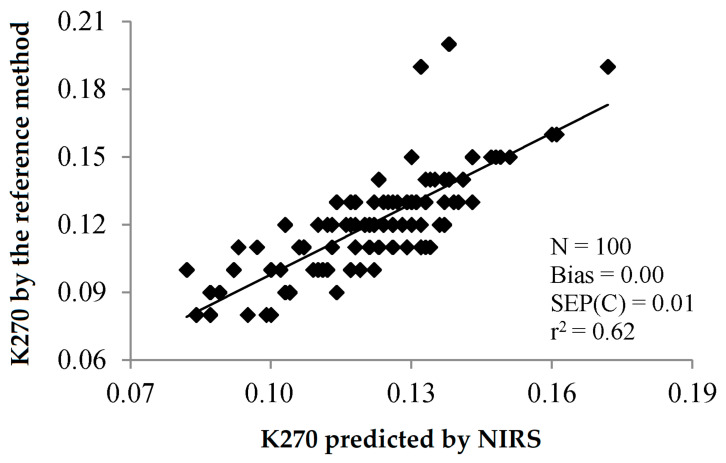
Validation exercise for the determination of K270 of olive oil by PLS-NIRS using all the wavelengths between 400 and 2500 nm [30].

The standard errors of laboratory for these methods have been reported to be 0.42 and 0.048 for K232 and k270, respectively [9]. These SEL have been calculated for ranges of values exceeding, by far, the limits established by the International Olive Council. Thus, SEL for K232 (0.42) was provided for samples in the range 1.7–20.4, while the maximum permitted value is 2.5. Similarly, SEL for K270 (0.048) was calculated for samples in the range 0.10–2.0, while the maximum permitted value is 0.2. This could make the comparison between the errors of prediction and SEL difficult. In any case, some of the statistics illustrated in Table 5 and Table 6 show the feasibility of using NIRS to determine K232 and K270. Reference [30] and, to a lesser extent, reference [47] show bias-corrected SEP and RMSEP, respectively, suitable for predicting the extinction coefficients by visible/NIR or NIR spectroscopy, respectively. Figure 11 and Figure 12 show the relation between K232 and 270 predicted by NIRS and K232 and 270 analysed by the reference method, as reported in Reference [30]. Similarly to what was observed for PV, the determination of the specific extinction coefficients by NIRS has lower precision than the determination of FA. It is noteworthy that all but one of the RPD values reported in Table 5 and Table 6 are less than 3, which accounts for the poor practical application of this parameter.

## 6. Other Compounds

In addition to the four basic quality physico-chemical parameters of olive oil, several other compounds and parameters of olive oil have been assayed to be quantified by NIRS (Table 7). The RPD values reported by the different authors on the various parameters are generally low, but RPD is not considered in the discussion of the statistics collected in Table 7 to the problems that its interpretation presents, as has been pointed out in the previous sections. For most of these parameters, the authors did not provide SEL, so the most suitable approach to assess the feasibility of NIRS to predict these parameters is then to compare the error of prediction with the unit range of these parameters for olive oils.

The UV absorbance at K225 is an index of oil bitterness. High bitterness in olive oils is not well accepted by consumers [18]. Using 13 PC, an R^2^_cal_ of 0.870 and an SEP of 0.058 were obtained for this parameter (Table 7), with SEL = 0.026 [18].

For carotenoid and chlorophyll pigments in VOO, Jiménez Marquez [17] concluded that his results showed similarities between visible-near infrared transmittance spectroscopy and reference laboratory methods. The SEL for chlorophylls was 0.25 mg/kg, while SEL for carotenes was 0.35 mg/kg for the ranges indicated in Table 7, with SEP being slightly superior to SEL [17]. The standard error of the laboratory depends on many factors. The range of concentrations used can be highlighted. In this sense, other authors have found that SEL was 0.23 mg/kg for carotenoids in the range 0.12–13.13 mg/kg, and 0.47 mg/kg for chlorophylls in the range 0.082–25.23 mg/kg [9]. Of note is that β-carotene is the precursor substance of vitamin A and is responsible for the yellow–orange colour of olive oil, while chlorophylls are responsible for the green colour of olive oil. Therefore, both absorb radiation mainly in the visible spectrum.

One could ask why one would determine these compounds by NIRS, since they absorb mainly in the visible spectrum and, as for the K225, K232 and K270 parameters, ultraviolet radiation. As stated in Section 3, the peaks observed at 420 and 460 nm in the olive oil spectrum correspond mainly to carotenoids, while the peak at approximately 670 nm corresponds to chlorophyll absorption [33]. This was the reason why the PLS models built solely with the NIR spectrum (Table 7) achieved maximum R^2^_cal_ of 0.66 and 0.56 for carotenoids and chlorophylls, respectively, which are too low for practical use [9,46]. For this reason, NIRS (or visible/NIR spectroscopy) should be implemented as a multiparametric tool, i.e., not only to determine a property of olive oil, but as many parameters as possible from its NIR (or visible/NIR) spectral information. The idea is to find out the composition and quality parameters of olive oil by simply acquiring its NIR or visible/NIR spectrum in a few seconds. This is the main advantage of NIRS when compared to the laborious, time-consuming reference methods that have to be individually carried out in the laboratory for each quality parameter of olive oil.

Alkyl esters in olive oils are derived from the non-desired fermentation of the fruit, normally when overripe or incorrectly stored, thus suffering damage in the cell structure prior to entering the olive oil processing. The most important quality of olive oil is the number of ethyl esters, which is regarded as a quality criterion by the International Olive Council. The content of fatty acid ethyl esters must be ≤35 mg/kg for an oil to be classified as EVOO [1]. The SEP illustrated in Table 7 for ethyl esters (14.2 mg/kg) [30] seems to be a bit excessive to meet the requirements of the International Olive Council.

**Figure 13:**
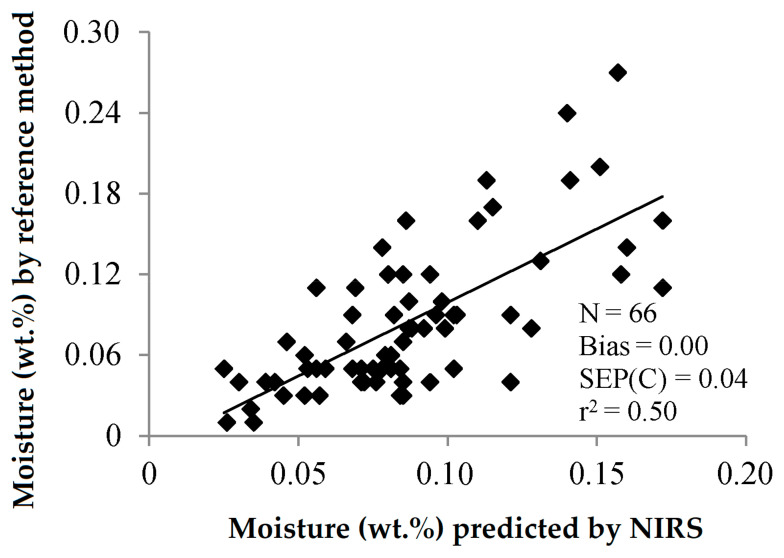
Validation exercise for the determination of the moisture and volatile matter content of olive oil by PLS-NIRS using all the wavelengths between 400 and 2500 nm [30].

Moisture, which can promote the rancidification of olive oil, leading to an unpleasant taste and an unpleasant odour, has been determined by PLS-NIRS to achieve an r^2^_cal_ of 0.71 and a bias-corrected SEP of 0.04 wt.% [30]. Taking into account that the moisture and volatile matter content is another quality criterion of the International Olive Council, and it must be ≤0.2 wt.% for edible olive oils [1], this prediction error should be lowered a bit. The relation between analysed and predicted values obtained by these authors is illustrated in Figure 13. It is worth noting that the determination of water content in olive oils by NIRS has not been assayed to date by using only the wavelengths where the broad absorption bands of water are found (at 1400 and 1950 nm).

Parameters such as total polyphenols have not been successfully predicted by NIRS. In this sense, SEPs of 82.10 and 89.66 mg/kg were obtained when analysing total polyphenols in EVOO using two spectrometers, SEL being 9.24 mg/kg for samples in the range 44.49–738.76 mg/kg [9]. Other authors achieved a good correlation coefficient of calibration (r^2^_cal_ = 0.85) and a lower error of prediction (RMSEP = 44.5 mg/kg) [47], but these were still a bit high for practical use.

Squalene is a hydrocarbon that can be found in relatively high quantities (between 60 and 75 wt.%) within the unsaponifiable fraction of olive oil, accounting for between 0.2 and 7.5 g/kg of olive oil [67]. In spite of the multiple pieces of scientific evidence of the beneficial effects of squalene on human health, its determination is generally not performed in the olive oil industry, as squalene is neither considered a quality nor a purity parameter in olive oil regulation [1]. The only attempt found in the literature to determine squalene in olive oil by NIRS or visible/NIR spectroscopy used EVOO, VOO, ordinary oil oils, pomace oils, and lampante oils of different varieties for the calibration and validation exercises [23]. The best results were obtained with the NIR spectra (Table 7). However, the SEP achieved (1 g/kg) is too high for its use in the olive oil industry since, as aforementioned, the concentration of squalene in olive oils ranges between 0.2 and 7.5 g/kg olive oil.

Olive oil is a notorious source of vitamin E (α-tocopherol). EVOO and VOO contain about 207.3 mg α-tocopherol per kg of olives. Pomace olive oils contain higher amounts of vitamin E, up to 981.6 mg/kg [68]. The determination of α-tocopherol, β-tocopherol, γ-tocopherol and total tocopherols of olive oils has been assayed using their NIR and visible/NIR spectra [25]. In that work, lampante and pomace olive oils were used in the calibration PLS models along with EVOO and VOO to increase the diversity of tocopherols, so that the range of concentrations of α-tocopherol (Table 7) was much higher than the content of α-tocopherol reported for EVOO and VOO [68]. Models using only NIR wavelengths predicted the content in α-, γ- and total tocopherols better than those using all wavelengths from the visible/VIR spectrum [25]. The PLS-NIR model for α-tocopherol achieved a good correlation coefficient of calibration (0.95), but SEP (47.2 mg/kg) seems to be quite high for practical application, taking into account that the average content of vitamin E in olive oils is 207.3 mg/kg [68]. The statistics found by other authors did not improve the ability of NIRS to determine α-tocopherol in olive oils [47].

Finally, other parameters of interest for the quality of olive oil, such as the oxidative stability, for which the units are time-based, have been predicted by visible/NIR spectroscopy with relatively good precision [27], as illustrated in Table 7.

On the other hand, olive oil is practically composed of fat (the saponifiable fraction accounts for roughly 98 wt.% olive oil). The fatty acid profile of olive oils is one of the most suitable and with the highest precision analysis that NIRS can perform [10,18,31,32,69]. The current European regulation settles the obligation of food manufacturers to include nutritional information on their product labels [26]. Mandatory information on food labels includes energy value, total fat content, total saturated fatty acids (TSFA), and other compounds that olive oil does not contain, such as carbohydrates, sugars, proteins, and salt. As voluntary nutritional information, the European label can contain other nutritional information, such as monounsaturated fatty acids (MUFA) and polyunsaturated fatty acid (PUFA) content. Furthermore, food labelling regulations in the USA and Canada also require a declaration of TSFA content on product labels [10]. Regarding olive oil, the most frequently included information on its nutritional label is total fat, saturated fat, monounsaturated fat, and polyunsaturated fat [26]. It has been reported that the first overtone of MUFA can be observed at 1724 and 1766 nm, with the combination bands at 2358 nm [29]. As for PUFA, 1660, 1698, and 1730 nm wavelengths have correlated with the first overtone, 1162 and 1212 nm with the second overtone, and 2136, 2176, 2224, 2310, 2348, and 2434 nm with combination bands [29]. Some works available in the literature have shown the feasibility of NIRS for determining TSFA, MUFA, and PUFA in the American, Canadian, Spanish, and Portuguese EVOO, VOO, and ordinary olive oils [10,14,26,47]. Hence, NIRS is suitable for the nutritional labelling of olive oil.

## 7. Sensory Attributes

The sensory parameters of olive oil are of equal importance as the physico-chemical quality parameters described in Section 5. Notwithstanding, scarce information can be found in the literature on the use of NIRS for the determination of the sensory parameters of olive oil. The prediction of the minor composition of VOO, in particular its phenolic and volatile compounds, as well as its organoleptic attributes, has been assayed in the 800–2500 nm NIR spectrum. Acceptable multivariate algorithms based on the multiple coefficient of determination were obtained for some minor components, such as hydroxytyrosol derivatives (r^2^ = 0.86–0.88) and C6 alcohols (r^2^ = 0.69–0.80), and for positive sensory attributes such as ‘fruity’ (r^2^ = 0.87) and ‘bitter’ (r^2^ = 0.85) [47]. More research is needed to correlate the NIR spectra of olive oil with its sensory parameters before regarding NIRS as a potential tool for the determination of these parameters.

## 8. Conclusions

The information available in the literature illustrates that the application of NIRS to olive oil could undergo an industrial development similar to that of olives and olive pastes, which have commercial, available NIRS equipment for assessing some of its main parameters of interest. A sampling system of olive oils and NIRS equipment for the acquisition of their NIR spectra could be implemented in the olive oil mill or in the bottling plant, thus allowing the on-site determination of their main quality parameters.

The four primary olive oils’ quality parameters (FA, PV, K232 and K270) can be accurately determined by NIRS spectroscopy, based on promising results reported by different authors. In addition, NIRS is suitable for the nutritional labelling of olive oil, since its feasibility for determining TSFA, MUFA, and PUFA has been demonstrated. Therefore, all these parameters in an olive oil could be measured by NIRS, as a multiparametric analytical technique, simply by acquiring the NIR spectrum of the oil and using the PLS model developed for each parameter.

Other parameters such as α-tocopherol (vitamin E), fatty acid ethyl esters, squalene and K225 show potential to be determined by NIRS, but the prediction errors reported by the various authors are still a bit high for practical application. Furthermore, by expanding the wavelength range to which spectra are acquired to the visible region, other compounds such as carotenoids and chlorophylls, which absorb radiation mainly in the visible spectrum, could be quantified by visible/NIR spectroscopy. However, visible/NIR spectrophotometers are more expensive than NIR spectrophotometers, so the implementation of one or another will depend on the robustness of the PLS models for each parameter and, hence, their practical application.

NIRS equipment at olive oil mills or bottling plants should provide self-learning model calibration systems, so that samples from new harvestings, different designations, geographical origin, and varieties, etc. are automatically added to the calibration set to strengthen the PLS models over time. Validation exercises with samples not used to build the PLS models are mandatory to assess their performance.

Spectra pre-treatments (derivatisation, normalisation, baseline correction, standard normal variate, mean centring, Savitzky and Golay smoothing, first and second derivatives, multiplicative scatter corrections) enhance the handling of the spectra and the building of the PLS calibration models. Similarly, the selection of actual contributing spectral variables and the removal of outliers can improve the performance of the PLS models. Notwithstanding, these latter two procedures must be carefully performed or avoided at early stages of the model building (when there is not a large calibration sample set) due to the risk of removing important spectral information related to the quality parameter of interest.

The ultimate goal is to achieve acceptance of NIRS as an official method for the determination of the quality parameters and the nutritional parameters for the labelling of olive oil by the relevant national authorities and, as a priority, the International Olive Council, which will greatly contribute to the industrial development of NIRS equipment for the olive oil industry.

## Figures and Tables

**Table 1:** Criteria for the assessment of PLS models in NIRS [52].

Calibration (r^2^_c_)		Prediction (SEP)	
r^2^_c_ ≥ 0.90	Excellent precision	SEP = 1–1.5 SEL	Excellent precision
r^2^_c_ = 0.70–0.89	Good precision	SEP = 2–3 SEL	Good precision
r^2^_c_ = 0.50–0.69	Good separation between low, medium, and high values	SEP = 4 SEL	Medium precision
r^2^_c_ = 0.30–0.49	Correct separation between low and high values	SEP = 5 SEL	Low precision
r^2^_c_ = 0.05–0.29	It is better than no analysing		

r^2^_c_: correlation coefficient of calibration; SEP: standard error of prediction; SEL: standard error of laboratory.

**Table 2:** PLS statistics obtained for free acidity (FA) of olive oils using different FA ranges, visible/NIR spectral intervals and optical path lengths.

FA Range (%)	Spectral Acquisition	Spectrum (nm)	n_cal_	n_val_	Path Length (mm)	PC	r^2^_cal_	SEP	RPD	Reference
0.25–4.5	Transmittance	1100–2500	72	35	0.2	7	0.69	0.12	1.8	[9]
0.25–4.5	Transflectance	978–2500	72	35	0.6	8	0.58	0.15	1.5	[9]
0.15–1.3	Reflectance	1961–2212	62	17	8.0	12	0.99	0.060	-	[12]
0.12–15.1	Transmittance	1100–2500	131	45	1.0	15	0.99	0.16	-	[18]
0.10–1.3	Transmittance	800–2200	87	40	10.0	15	0.94	0.075	2.6	[22]
0.16–0.5	Reflectance	1100–2300	38	19	-	-	0.89	0.023	-	[24]
0.10−8.7	Transmittance	350–2500	222	47	5.0	-	0.86	0.35	3.1	[27]
0.10–5.7	Transflectance	400–2500	359	100	0.1	-	0.99	0.060 ^1^	7.7	[30]
0.06–8.0	Transmittance	400–2250	208	-	-	-	0.97	0070 ^2^	8.4	[32]
0.10–1.1	Transmittance	800–2500	60	37	8.0	-	0.76	0.080 ^3^	1.5	[47]
0.36–3.3	Transmittance	400–1100	34	14	3.0	2	0.88	0.34 ^3^	3.1	[61]
0.11–1.7	Transmittance	800–2500	14	10	6.5	13	0.99	0.048 ^3^	-	[62]
0.15–2.2	Transmittance	1000–2222	49	11	-	8	0.98	0.088 ^4^	4.9	[63]

n_cal_ = number of samples in the calibration set; n_val_ = number of samples in the validation set; PC = number of principal components; r^2^_c_ = multiple correlation coefficient of calibration; SEP = standard error of validation; RPD = ratio of performance to deviation. ^1^ bias-corrected standard error of prediction; ^2^ standard error of cross validation; ^3^ root mean square error of prediction; ^4^ root mean square error of cross validation.

**Table 3:** Spectral bands’ tentative assignments correlated to FA of olive oil obtained by the Monte Carlo uninformative variable elimination (MCUVE) method and the successive projections algorithm (SPA).

MCUVE (nm)	SPA (nm)	Bond	Vibration	Molecule/Compound	Reference
1202–1221	-	C–H	First overtone	–CH=CH–	[29]
		C–H	Second overtone	–CH_2_	[65]
1484–1506	-	O–H stretch	First overtone	Cellulose	[65]
1531–1569	-	O–H stretch	First overtone	Starch	[65]
1582–1603	-	-	-	-	-
1613–1644	-	C–H stretch	First overtone	=CH_2_	[65]
-	1717–1729	C–H stretch	First overtone	–CH_3_	[29,65]
		C–H stretch	First overtone		
-	1751–1763	C–H stretch	First overtone	–CH_2_	[29,65]
1915–1934	-	C=O stretch	Second overtone	CONH	[65]
1957–1973	-	O–H stretch	O–H bend combination	Starch and cellulose	[65]
2154–2192	-	C–H Asym C–H stretch C–H stretch	Combination	–CH_2_	[29]
			C–H deformation combination	–HC=CH–	[65]
			C=O stretch combination	Protein	[65]
			Combination bands	–COOH	[66]

**Table 4:** PLS statistics obtained for peroxide value (PV) of olive oils using different PV ranges, visible/NIR spectral intervals, and optical path lengths.

PV Range (meq O_2_/kg)	Spectral Acquisition	Spectrum (nm)	n_cal_	n_val_	Path Length (MM)	PC	R^2^_cal_	SEP	RPD	Reference
2.2–74.0	Transmittance	1100–2500	90	44	0.2	6	0.92	4.15	3.5	[9]
2.2–74.0	Transflectance	1100–2500	90	44	0.6	8	0.87	5.28	2.8	[9]
3.0–32.0	Reflectance	1333–1587	65	14	8.0	12	0.98	1.0	-	[12]
5.6−43.9	Transmittance	350–2500	199	46	5.0	-	0.87	3.82	2.8	[27]
1.6–44.5	Transflectance	400–2500	359	100	0.1	-	0.83	1.31 ^1^	2.0	[30]
2.6–18.0	Transmittance	400–2250	125	-	-	-	0.92	1.34 ^2^	2.7	[32]
7.1–75.4	Transmittance	800–2500	60	37	8.0	-	0.92	2.65 ^3^	1.6	[47]
3.6–8.0	Transmittance	400–1100	34	14	3.0	2	0.83	2.25 ^3^	3.1	[61]
2.5–17.2	Transmittance	800–2500	14	10	6.5	10	0.94	1.87 ^3^	-	[62]
0.0–26.7	Transmittance	1000–2222	49	11	-	8	0.84	3.0 ^4^	1.8	[63]

n_cal_ = number of samples in the calibration set; n_val_ = number of samples in the validation set; PC = number of principal components; r^2^_c_, = multiple correlation coefficient of calibration; SEP = standard error of validation; RPD = ratio of performance to deviation. ^1^ bias-corrected standard error of prediction; ^2^ standard error of cross validation; ^3^ root mean square error of prediction; ^4^ root mean square error of cross validation.

**Table 5:** PLS statistics obtained for K232 of olive oils using different K232 ranges, visible/NIR spectral intervals, and optical path lengths.

K232 (AU)	Spectral Acquisition	Spectrum (nm)	n_cal_	n_val_	Path Length (mm)	PC	r^2^_cal_	SEP	RPD	Reference
1.7–20.4	Transmittance	1100–2500	70	34	0.2	6	0.94	0.94	3.6	[9]
1.7–20.4	Transflectance	978–2500	70	34	0.6	4	0.87	1.3	2.6	[9]
0.9−5.0	Transmittance	350–2500	223	55	5.0	-	0.82	0.32	2.6	[27]
1.4–5.4	Transflectance	400–2500	359	100	0.1	-	0.75	0.10 ^1^	1.5	[30]
1.2–2.0	Transmittance	800–2500	60	37	8.0	-	0.40	0.090 ^2^	1.2	[47]
1.5–3.5	Transmittance	1000–2222	49	11	-	8	0.84	0.27 ^2,3^	1.6	[63]

n_cal_ = number of samples in the calibration set; n_val_ = number of samples in the validation set; PC = number of principal components; r^2^_c_ = multiple correlation coefficient of calibration; SEP = standard error of validation; RPD = ratio of performance to deviation. ^1^ bias-corrected standard error of prediction; ^2^ root mean square error of prediction; ^3^ root mean square error of cross validation.

**Table 6:** PLS statistics obtained for K270 of olive oils using different K270 ranges, visible/NIR spectral intervals and optical path lengths.

K270 (AU)	Spectral Acquisition	Spectrum (nm)	n_cal_	n_val_	Path Length (mm)	PC	r^2^_cal_	SEP	RPD	Reference
0.10–2.0	Transmittance	1100–2500	70	34	0.2	6	0.87	0.094	2.5	[9]
0.10–2.0	Transflectance	978–2500	70	34	0.6	3	0.71	0.13	1.8	[9]
0.07–0.41	Transflectance	400–2500	359	100	0.1	-	0.67	0.012 ^1^	2.2	[30]
0.06–0.17	Transmittance	800–2500	60	37	8.0	-	0.54	0.020 ^2^	1.2	[47]
0.08–0.21	Transmittance	1000–2222	49	11	-	10	0.74	0.019 ^3^	1.6	[63]

n_cal_ = number of samples in the calibration set; n_val_ = number of samples in the validation set; PC = number of principal components. ^1^ bias-corrected standard error of prediction; ^2^ root mean square error of prediction; ^3^ root mean square error of cross-validation.

**Table 7:** PLS statistics obtained for different compounds and parameters of olive oils using different unit ranges, visible/NIR spectral intervals, and optical path lengths.

Parameter	Units	Range	Spectral Acquisition	Spectrum (nm)	n_cal_	n_val_	Path Length (mm)	PC	r^2^_cal_	SEP	RPD	Reference
K225	AU	0.06–0.66	Transmittance	1100–2500	149	30	1.0	13	0.87	0.058	-	[18]
Carotenoids	mg/kg	1.6–18.1	Transmittance	450–2500	151	32	1.0	4	0.985	0.66	-	[17]
Carotenoids	mg/kg	0.12–13.1	Transmittance	1100–2500	64	32	0.2	5	0.66	1.1	1.7	[9]
Carotenoids	mg/kg	0.12–13.1	Transflectance	978–2500	64	32	0.6	3	0.52	1.4	1.4	[9]
Carotenoids	mg/kg	2.1–38.5	Transmittance	1100–2500	205	50	5.0	-	0.62	-	-	[46]
Carotenoids	mg/kg	2.1–38.5	Transmittance	350–2500	205	50	5.0	-	0.95	1.8	3.9	[46]
Chlorophylls	mg/kg	0.7–27.5	Transmittance	450–2500	151	32	1.0	4	0.993	0.96	-	[17]
Chlorophylls	mg/kg	0.082–25.2	Transmittance	1100–2500	65	32	0.2	8	0.56	3.6	1.5	[9]
Chlorophylls	mg/kg	0.082–25.2	Transflectance	978–2500	65	32	0.6	3	0.31	4.4	1.2	[9]
Chlorophylls	g/kg	0–14.5	Transmittance	400–2250	168	-	-	-	0.98	0.51 ^1^	5.6	[32]
Chlorophylls	mg/kg	1.4–88.1	Transmittance	1100–2500	205	53	5.0	-	0.56	-	-	[46]
Chlorophylls	mg/kg	1.4–88.1	Transmittance	350–2500	205	53	5.0	-	0.96	3.5	4.1	[46]
Alkyl esters	mg/kg	3–610	Transflectance	400–2500	359	100	0.1	-	0.79	19.5 ^2^	1.9	[30]
Ethyl esters	mg/kg	1–461	Transflectance	400–2500	359	100	0.1	-	0.80	14.2 ^2^	1.9	[30]
Moisture	wt.%	0.01−0.63	Transflectance	400–2500	283	66	0.1	-	0.71	0.04 ^2^	1.5	[30]
Total polyphenols	mg/kg	44.5–738.8	Transmittance	1100–2500	67	31	0.2	2	0.21	89.7	1.1	[9]
Total polyphenols	mg/kg	44.5–738.8	Transflectance	978–2500	67	31	0.6	2	0.34	82.1	1.2	[9]
Total polyphenols	mg/kg	110.7–594.0	Transmittance	800–2500	60	37	0.8	-	0.85	44.5 ^3^	1.7	[47]
Squalene	g/kg	1.0−10.1	Transflectance	1100–2300	118	59	-	-	0.86	1.2	2.3	[23]
Squalene	g/kg	1.0−10.1	Transmittance	350–2500	118	59	10.0	-	0.76	1.0	1.9	[23]
α-tocopherol	mg/kg	54.5–755.9	Transflectance	1100–2300	218	109	10.0		0.95	47.2	2.4	[25]
α-tocopherol	mg/kg	54.5–755.9	Transmittance	350–2500	218	109	10.0		0.94	58.3	1.9	[25]
α-tocopherol	mg/kg	91.0–249.3	Transmittance	800–2500	60	37	0.8	-	0.71	15.2 ^3^	1.3	[47]
β-tocopherol	mg/kg	0.5–14.1	Transflectance	1100–2300	218	109	10.0		0.64	1.4	1.0	[25]
β-tocopherol	mg/kg	0.5–14.1	Transmittance	350–2500	218	109	10.0		0.66	1.3	1.1	[25]
β-tocopherol	mg/kg	9.11–17.2	Transmittance	800–2500	60	37	0.8	-	0.42	1.5 ^3^	1.0	[47]
γ-tocopherol	mg/kg	1.8–103.8	Transflectance	1100–2300	218	109	10.0		0.92	6.3	1.9	[25]
γ-tocopherol	mg/kg	1.8–103.8	Transmittance	350–2500	218	109	10.0		0.87	8.1	1.5	[25]
γ-tocopherol	mg/kg	10.7–36.6	Transmittance	800–2500	60	37	0.8	-	0.63	2.2 ^3^	1.2	[47]
Total tocopherol	mg/kg	63.1–1078.0	Transflectance	1100–2300	218	109	10.0		0.92	61.8	2.0	[25]
Total tocopherol	mg/kg	63.1–1078.0	Transmittance	350–2500	218	109	10.0		0.91	76.2	1.6	[25]
Total tocopherol	mg/kg	110.8–278.8	Transmittance	800–2500	60	37	0.8	-	0.61	19.3 ^3^	1.2	[47]
Oxidative stability	h	15.2−90.6	Transmittance	350–2500	133	43	5.0	-	0.94	7.4	3.0	[27]

n_cal_ = number of samples in the calibration set; n_val_ = number of samples in the validation set; PC = number of principal components; r^2^_c_ = multiple correlation coefficient of calibration; SEP = standard error of validation; RPD = ratio of performance to deviation. ^1^ standard error of cross validation; ^2^ bias-corrected standard error of prediction; ^3^ root mean square error of prediction.
